# 茶多酚对小鼠Lewis肺癌移植瘤中NF-κB、COX-2、Survivin表达的影响

**DOI:** 10.3779/j.issn.1009-3419.2012.05.04

**Published:** 2012-05-20

**Authors:** 婧 王, 信义 陈, 丽 侯, 莉 李, 鸿飞 陆, 伟 刘

**Affiliations:** 1 100700 北京，北京中医药大学东直门医院肿瘤血液科 Department of Oncology and Hematology, Dongzhimen Hospital Affiliated to Beijing University of Chinese Medicine, Beijing 100700, China; 2 100700 北京，教育部、北京市重点实验室中医内科学 Ministry of Education and Beijing Municipal Key Laboratory, Chinese Internal Medicine Laboratory, Beijing 100700, China; 3 100102 北京，中国中医科学院望京医院 Wangjing Hospital, Chinese Academy of Traditional Chinese Medicine, Beijing 100102, China

**Keywords:** 茶多酚, 肺肿瘤, NF-κB, COX-2, Survivin, Tea polyphenols, Lung neoplasms, NF-κB, COX-2, Survivin

## Abstract

**背景与目的:**

肺癌是全球第一大恶性肿瘤，前期研究表明茶多酚具有一定的抗肺癌新生血管生成作用，本研究旨在观察茶多酚对小鼠Lewis肺癌移植瘤中NF-κB、COX-2、Survivin表达的影响，进而探讨茶多酚抗新生血管生成的效应机制。

**方法:**

建立C57BL/6小鼠肺癌移植瘤模型，测定模型对照组、沙利度胺组、茶多酚组以及茶多酚联合沙利度胺组的肿瘤抑制率，并且采用免疫组化法检测各组NF-κB、COX-2、Survivin表达水平，以探讨其抗肿瘤的分子机制。

**结果:**

实验表明，茶多酚具有如下作用：①沙利度胺组、茶多酚组以及茶多酚联合沙利度胺组的肿瘤抑制率分别为17.26%、20.81%和44.32%，茶多酚联合沙利度胺组与模型组瘤重比较，差异具有统计学意义（*P* < 0.05）；②NF-κB表达在茶多酚组及联合用药组有所降低，与模型对照组相比，联合用药组NF-κB表达明显下降，差异具有统计学意义（*P* < 0.05）；③COX-2表达在各治疗组均有所下降，与模型对照组相比，联合用药组表达明显下降（*P* < 0.05）；④Survivin表达在各治疗组均较模型对照组明显降低（*P* < 0.05），其中茶多酚组下降最为明显（*P* < 0.01）。

**结论:**

茶多酚联合沙利度胺组对肺癌有明显抑制作用，其机制可能与抑制NF-κB信号通路的异常激活、抑制NF-κB活化、降低COX-2表达、并降低内皮细胞Survivin表达从而抗肺癌新生血管生成相关。

## Abstract (en)

**Background and objective:**

Lung cancer is the leading cause of cancer-related deaths worldwide. The anti-angiogenic effect of tea polyphenols (TPs) on lung carcinoma was confirmed in our previous study. The effect of TPs on the expression of nuclear factor kappa-light-chain-enhancer of activated B cells (NF-κB), cyclooxygenase-2 (COX-2), and Survivin in Lewis lung cancer xenograft in mice was observed to explore the anti-angiogenic mechanism of TPs.

**Methods:**

Lewis lung carcinoma was successfully implanted in 32 C57BL/6 mice, which were then randomly divided into the model control group, the Thalidomide group, the TPs group and the TPs combined with Thalidomide group. The tumor inhibition rates in these groups were determined, whereas the expressions of NF-κB, COX-2 and Survivin were detected using immunohistochemical techniques to investigate the molecular mechanism of the anti-tumor effect of TPs.

**Results:**

(1) The tumor inhibition rates in the Thalidomide control group, the TPs group and the TPs plus Thalidomide group were 17.26%, 20.81%, 44.32% respectively. Compared with the model control group, the tumor inhibition rate was significantly higher in the TPs plus Thalidomide group (*P* < 0.05); (2) Compared with the model control group, the expression of NF-κB was significantly decreased in the TPs group and the TPs plus Thalidomide group, and it was significantly downregulated in the latter (*P* < 0.05); (3) The expression of COX-2 decreased in all treatment groups, and was significantly downregulated in the TPs plus Thalidomide group compared with the model control group (*P* < 0.05); (4) Compared with the model control group, Survivin expression was significantly downregulated in all treatment groups (*P* < 0.05), and was most obviously reduced in the TPs plus the Thalidomide group (*P* < 0.01).

**Conclusion:**

TPs plus Thalidomide significantly inhibits Lewis lung cancer growth. TPs possibly downregulates the expression of NF-κB, COX-2 and Survivin in tumor tissue, thereby playing important roles in anti-angiogenesis.

肺癌是世界范围内肿瘤死亡的首要原因，约占肺癌80%的非小细胞肺癌（non-small cell lung cancer, NSCLC）在确诊时大多已属晚期，失去手术根治机会，临床以化、放疗等综合治疗为主。但目前化疗的疗效似乎到达了一个平台，其客观有效率约30%，中位生存期8个-10个月，1年生存率30%-40%，且不良反应较严重，多数患者难以完成有效的治疗周期。因此，探求新的治疗方法及策略对于提高NSCLC疗效尤为重要，抗肺癌新生血管生成即是目前主要研究方向之一。中医药在治疗NSCLC方面具有一定优势，前期研究^[1-4]^表明，绿茶的生物活性成分茶多酚（tea polyphenols, TPs）具有抗肿瘤效果，其机制可能与抑制肿瘤新生血管生成密切相关。

广泛存在于真核生物中的核因子-κB（nuclear factor-κB, NF-κB）是一种多功能的核转录因子；NF-κB信号传导通过控制多种细胞因子和生存基因表达，对恶性肿瘤细胞的增殖、生存、转移和血管生成具有一定的促进作用^[5, 6]^。环氧化酶（cyclooxygenase-2, COX-2）和Survivin是与NF-κB信号通路相关的重要蛋白分子。研究表明，肿瘤在缺氧状态下促血管生成因子COX-2、VEGF等的表达受NF-κB调控。肿瘤特异性凋亡抑制因子Survivin在肿瘤内皮细胞中也持续表达。通过免疫方法抑制内皮细胞中的Survivin表达可明显抑制肿瘤血管的生成，且大多数血管生成与抑制因子对肿瘤内皮细胞的作用都与调节Survivin的表达有关；而Survivin的表达主要是由AKT-NFκB来调节的，因此NF-κB信号通路可能是肿瘤血管生成的主要通路之一。本研究以NF-κB信号通路为主线，检测茶多酚对小鼠Lewis肺癌移植瘤中NF-κB、COX-2、Survivin表达的影响。

## 1 材料与方法

### 1.1 材料

#### 1.1.1 动物及细胞系

C57BL/6小鼠32只，6周-8周龄，SPF级，雌雄各半，由中国医学科学院肿瘤研究所肿瘤医院实验动物室繁育并提供。动物生产合格证号：京动许字（1999）第015号；动物合格证号：SCXK京2009-0007，在动物实验中心屏蔽系统辅以洁净层流柜环境中饲养。Lewis肺癌细胞系，由中国医学科学院肿瘤研究所检测中心常规保存并提供。

#### 1.1.2 药物

茶多酚由江西绿康天然产物有限责任公司惠赠，纯度98%；沙利度胺片为常州制药厂有限公司产品，国药准字H32026129，产品批号10060512。

#### 1.1.3 仪器与试剂

超净工作台系苏州宏瑞净化科技有限公司生产（型号SCW-CJ）；实验用离心机系江苏恒瑞制药机械有限公司生产（型号LS150）；倒置显微镜系日本NIKON株式会社生产（型号TS100）；电热恒温培养箱系黄石市恒丰医疗器械有限公司生产（型号SKP-02.420）；包埋机系天津航空机电公司生产（型号BMJ-1）；石蜡切片机系天津航空机电公司生产（型号QPJ-1C）；奥林巴斯显微镜BX51。兔抗NF-κB抗体、兔抗COX-2抗体、兔抗Survivin抗体、兔SP检测试剂盒及DAB显色试剂盒等购自武汉博士德生物工程有限公司。

### 1.2 方法

#### 1.2.1 细胞复苏与接种

液氮罐中取出含有Lewis肺癌细胞的冻存管，放入37 ℃水浴内，使其溶化，吸取细胞悬液，加入离心管并滴加培养液至10 mL，1, 000 r/min离心5 min，去上清液，加入PBS液后细胞计数，调整细胞悬液浓度至1×10^7^个/mL，以0.2 mL接种于小鼠右腋窝皮下，待肿瘤长至体积约为1, 000 mm^3^时移植。

#### 1.2.2 造模及分组

选择生长良好、瘤块无破溃的荷瘤小鼠，脱颈处死，无菌条件下剥离瘤块，用生理盐水洗去血渍，剪开瘤块，清除中心坏死组织，充分匀浆制备瘤细胞悬液，加生理盐水稀释细胞数至1×10^7^个/mL。取0.2 mL/只（含细胞数2×10^6^/只）。接种于小鼠右腋窝皮下，接种在30 min内完成。接种后第2天，根据性别分层，按肿瘤体积大小随机分为模型对照组、沙利度胺组、茶多酚组、茶多酚联合沙利度胺组，共4组，每组8只，称体重标号。

#### 1.2.3 给药剂量及方法

给药剂量均采用人与动物剂量换算法确定。①模型对照组，生理盐水每天0.2 mL/10 g灌胃；②沙利度胺对照组，沙利度胺按38 mg/kg/d（成人常用治疗量600 mg/70 kg/d换算）给药，生理盐水配制成浓度3.8 mg/mL，每天0.2 mL/10 g灌胃；③茶多酚组，茶多酚按337.5 mg/kg/d（成人治疗量2, 700 mg/70 kg/d换算）给药，生理盐水配置浓度为16.875 mg/mL，每天0.2 mL/10 g灌胃；④联合给药组，两药的给药剂量与单用药组相同，为保证给药剂量与灌胃体积的一致性，茶多酚配制浓度为675.0 mg/mL；沙利度胺配制浓度为7.6 mg/mL，每天各0.1 mL/10 g，灌胃。分组当天开始给药，每天1次，连续10天。

#### 1.2.4 瘤重及肿瘤抑制率

在实验过程中观察小鼠一般情况，用药结束后第2天处死小鼠，取瘤称重，并按如下公式计算肿瘤抑制率：抑制率（%）=[1-（治疗组平均瘤重/对照组平均瘤重）]×100%。

#### 1.2.5 免疫组化标本制备

取瘤称重后，每组随机选择6只小鼠移植瘤，制备肿瘤组织标本（约1 cm×1 cm×1 cm大小），用10%中性缓冲福尔马林液在室温或4 ℃条件下固定，在60 ℃以下环境内经脱水、透明、浸蜡及包埋制成蜡块。

#### 1.2.6 免疫组化指标检测

组织切片常规脱蜡、水化后，按相关试剂盒说明检测NF-κB、COX-2、Survivin蛋白表达。其中，NF-κB p65采用抗原修复液进行修复，抗体稀释度为1:30；COX-2进行酶修复，抗体稀释度为1:40；Survivin采用微波修复，抗体稀释度为1:30。结果判断：NF-κB、COX-2、Survivin蛋白阳性反应为棕黄色或棕褐色颗粒，NF-κB、Survivin主要定位于胞核或胞浆，COX-2主要定位于胞浆。各组每个指标选取6个肿瘤组织切片，每片选3个视野，用美国IMAGE-PRO 6.2图像分析系统作图像分析，测定光密度（integrated optical density, IOD）值。

#### 1.2.7 统计学处理

所有数据用Mean±SD表示，采用SPSS 13.0统计软件进行数据处理。采用单因素方差分析*SNK*检验或*Tamhane’s T2*检验进行组间比较。*P* < 0.05为差异有统计学意义。

## 2 结果

### 2.1 瘤重及肿瘤抑制率

沙利度胺组、茶多酚组、联合用药组的肿瘤重量均低于对照组，提示对肿瘤具有抑制作用。其中，茶多酚联合沙利度胺组的肿瘤抑制率为44.32%，与对照组相比，差异具有统计学意义（*P* < 0.05）。联合用药组抑瘤率与沙利度胺组、茶多酚组比较，无明显差别（*P* > 0.05）。结果见表 1。

**1:** 实验各组肿瘤重量及抑瘤率的比较（Mean±SD） Comparision of tumor weight and tumor inhibition rate in each group (Mean±SD)

Group	*n*	Tumor weight (g)	*P*	Tumor inhibition rate (%)
Model control group	8	1.550±0.808	-	-
Thalidomide group	8	1.283±0.432	0.331	17.26
TPs group	8	1.228±0.766	0.241	20.81
TPs plus Thalidomide group	8	0.863±0.353^*^	0.014^*^	44.32
Compared with model group, ^*^P < 0.05. TPs: tea polyphenols.				

### 2.2 NF-κB、COX-2、Survivin表达

实验各组NF-κB、COX-2、Survivin表达检测结果见表 2。由表 2可知，NF-κB表达在茶多酚组及联合用药组有所降低，与模型对照组相比，联合用药组NF-κB表达降低明显，差异具有统计学意义（*P* < 0.05）；且联合用药组NF-κB表达低于沙利度胺组（*P* < 0.05），与茶多酚组相比无明显差异（*P* > 0.05）。COX-2表达在各治疗组均有所下降，与模型对照组相比，联合用药组表达明显下降（*P* < 0.05）；且联合用药组COX-2表达明显低于沙利度胺组（*P* < 0.05）和茶多酚组（*P* < 0.01）。而Survivin表达在各治疗组均较模型对照组明显降低（*P* < 0.05），其中茶多酚组下降最为明显（*P* < 0.01）；茶多酚组Survivin表达较联合用药及沙利度胺组下降更为明显（*P* < 0.01）。各实验组NF-κB、COX-2、Survivin表达见图 1，其中NF-κB阳性表达主要为胞核棕染，COX-2、Survivin阳性表达主要为胞质棕染。

**2:** 各实验组NF-*κ*B、COX-2和Survivin表达的IOD值比较（Mean±SD） Comparision of IOD value of NF-*κ*B, COX-2 and Survivin in each group (Mean±SD)

Group	NF-*κ*B (×10^4^)	COX-2 (×10^4^)	Survivin
Model control group	85.59±31.23	275.92±77.02	168.58±41.31
Thalidomide group	90.11±40.73^∆^	111.07±43.10^∆^	106.82±68.30^*^
TPs group	54.75±22.49	131.19±43.99^∆∆^	52.97±22.96^*^^*^^∆∆^
TPs plus Thalidomide group	46.15±16.32^*^	60.03±26.29^*^	116.15±31.25^*^
Compared with model group: ^*^P < 0.05, ^**^P < 0.01; Compared with drug combination group: ^∆^*P* < 0.05, ^∆∆^*P* < 0.01. IOD: integrated optical density.			

**1:**
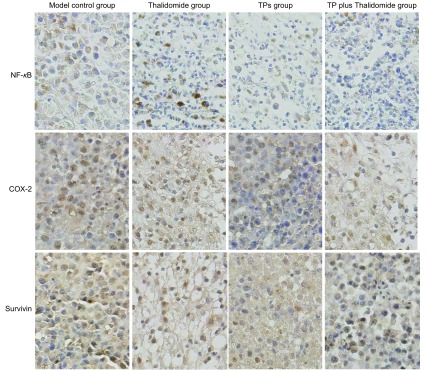
各实验组NF-*κ*B、COX-2和Survivin表达水平比较（SP, ×400） Expressions of NF-*κ*B, COX-2 and Survivin in all groups (SP, ×400)

## 3 讨论

随着研究的不断深入，抗新生血管药物在非小细胞肺癌临床治疗中发挥了越来越重要的作用。目前世界上处于研究和开发中的肿瘤血管生成抑制候选物超过300种，其中有数十种进入临床研究阶段。以贝伐珠单抗（Avastin）、重组人血管内皮抑制素（Endostar）、沙利度胺（Thalidomide）等为代表的抑制肺癌新生血管药物虽然显示了一定的治疗效果，却存在如下问题：首先，Endostar在体内代谢快，并有发生心肌缺血的危险性，对高龄合并有心脑血管疾病的患者应权衡患者治疗受益和弊端。Thalidomide副作用相对较多，除常见的嗜睡、疲倦、便秘等副作用外，也可见周围神经病、深静脉血栓、中毒性表皮坏死溶解症等较重副作用，严重影响其临床应用；其次，Avastin、Endostar合成及提取费用昂贵，患者持续性用药会明显增加患者经济负担，限制了临床推广应用；再者，肿瘤血管生成是多因素作用的结果，而这些药物大都只针对肿瘤血管生成诸多环节中的单一途径，临床疗效并不如实验结果那样理想和切实可行。因此从天然植物或中草药中寻找新型抗肿瘤血管生成药物成为当前肿瘤治疗研究的热点和突破口。

茶叶具有很好的药用价值，唐代即有“茶药”一词。《唐本草》也指出，茶叶能“清头目，除烦渴，化痰，消食，利尿，解毒”。近些年临床工作者将其“化痰与解毒”的功能拓展到应用于恶性肿瘤的临床治疗。现代研究表明，其抗肿瘤的主要成分是茶叶中生物活性成分茶多酚。茶多酚由30多种含酚基的物质组成，按化学结构可分为4类：儿茶素类、黄酮及黄酮醇类、花白素和花青素类、酚酸类，其中以儿茶素含量最高，占总量的60%-80%，主要由D, L-C、EC、EGC、EGCE、GCG、ECG等几种单体组成。前期研究^[1-4]^表明，茶多酚可下调VEGF、bFGF表达、上调TIMP-2表达并降低肿瘤组织MVD，联合化疗可以提高NSCLC的疗效，但是对其所经过的信号通路、作用机制与靶点尚不明确。

NF-κB是一种细胞核转录因子，因其能够与B细胞免疫球蛋白的κ亲链基因的增强子κB序列（GGGACTTTCC）特异性结合而得名。在未受刺激时，NF-κB二聚体与抑制性蛋白IκB结合，形成NF-κB/IκB复合物，存在于胞质中；外源性刺激通过信号转导通路激活IκB激酶（IKK）上游的激酶而使IKK活化，活化的IKK再使其底物IκB磷酸化，并进一步降解，NF-κB发生核异位，与靶基因的特异位点相结合，从而调控靶基因的转录^[7]^。IKK/IκB/NF-κB途径各成员的异常有可能影响NF-κB的组成性活化，使NF-κB呈高水平诱导性活化，这在肿瘤的发生、转移和血管生成过程中具有重要意义。COX-2是前列腺素（prostaglandins, PGs）合成过程中的重要限速酶，许多肿瘤组织COX-2表达水平很高，而在正常组织中不表达或表达很低^[8]^。COX-2的启动子序列中含有NF-κB特异结合序列，该序列与NF-κB结合后可以促进COX-2基因的转录；COX-2又可通过上调VEGF表达参与肿瘤血管生成^[9]^。Survivin是凋亡抑制蛋白（inhibitor of apoptosis protein, IAP）家族的成员，其过度表达抑制了细胞凋亡，从而促进细胞异常增殖与恶性转化。近年研究^[10]^发现Survivin在内皮细胞中表达，与肿瘤血管生成密切相关；并且在NSCLC中，COX-2表达与Survivin表达呈正相关，而反义COX-2衍生的癌细胞中Survivin水平明显降低^[11]^。

在前期实验中，我们发现茶多酚对肺癌新生血管生成具有一定抑制作用：与模型对照组相比，沙利度胺组、茶多酚组、茶多酚联合沙利度胺组的肺癌移植瘤微血管密度（micro-vessel density, MVD）明显降低（*P* < 0.01），联合用药组下降较单药组更为明显（*P* < 0.05）；并且VEGF表达在用药各组较模型对照组明显降低（*P* < 0.01）。这初步证明，茶多酚具有抗肺癌新生血管生成的效应。

本实验在建立C57小鼠Lewis肺癌移植瘤的基础上，采用免疫组化方法观测NF-κB信号通路的相关因子表达，进而探讨茶多酚抗肺癌新生血管生成的可能效应机制及靶点。实验中的阳性对照药物沙利度胺又称反应停，是抑制新生血管生成的代表药物，其治疗机理与抑制血管内皮生长因子bFGF和VEGF的表达、抑制TNF-α的分泌、降低血管内皮细胞整合素等相关^[12]^。本项研究结果表明，茶多酚联合沙利度胺能够明显抑制肿瘤生长，结合前期实验结果，其作用可能与抗肿瘤新生血管生成相关。而其抑制肿瘤新生血管的效应机制，则可能与抑制NF-κB信号通路的异常激活、抑制NF-κB活化、降低COX-2表达并降低内皮细胞Survivin表达相关。联合应用茶多酚与沙利度胺对NF-κB和COX-2表达的抑制作用优于单独用药，二者具有协同作用。除NF-κB信号通路外，茶多酚抑制Survivin表达尚存在其它主要途径，但未能提高茶多酚抗肿瘤效应。通过研究我们发现，茶多酚在肿瘤治疗中具有广泛的应用前景。肿瘤的发生是多步骤、多环节作用的结果，我们还将从不同层次探索茶多酚抗肺癌作用靶点，揭示茶多酚抗肺癌机制，为推广应用及新药研发提供理论依据。
